# Syndrome of Extreme Insulin Resistance (Rabson-Mendenhall Phenotype) with Atrial Septal Defect: Clinical Presentation and Treatment Outcomes

**DOI:** 10.4274/Jcrpe.857

**Published:** 2013-03-21

**Authors:** Deep Dutta, Indira Maisnam, Sujoy Ghosh, Satinath Mukhopadhyay, Subhankar Chowdhury

**Affiliations:** 1 IPGMER & SSKM Hospital, Department of Endocrinology & Metabolism, Kolkata, India

**Keywords:** Rabson-Mendenhall syndrome, insulin resistance, lipoatrophy, atrial septal defect, short stature

## Abstract

Syndrome of extreme insulin resistance (SEIR) is a rare spectrum disorder with a primary defect in insulin receptor signalling, noted primarily in children, and is often difficult to diagnose due to the clinical heterogeneity. SEIR was diagnosed in an adolescent girl with facial dysmorphism, exuberant scalp and body hair, severe acanthosis, lipoatrophy, dental abnormalities, and short stature (Rabson-Mendenhall phenotype). She had elevated fasting (422.95 pmol/L) and post-glucose insulin levels (>2083 pmol/L). Total body fat was decreased (11%; dual-energy X-ray absorptiometry). Basal growth hormone (GH) was increased (7.9 μg/L) with normal insuline-like growth factor 1 (37.6 nmol/L) suggestive of GH resistance. She had fatty liver and polycystic ovaries. Echocardiography revealed ostium secundum type atrial septal defect (ASD). Blood glucose normalized with pioglitazone (30 mg/day). Delayed development, severe insulin resistance, mild hyperglycemia, absence of ketosis, and remarkable response of hyperinsulinemia and hyperglycemia to pioglitazone which persisted even after 1 year of diagnosis are some of the notable features of this patient. This is perhaps the first report of occurrence of congenital heart disease (ASD) in a patient of SEIR (Rabson-Mendenhall phenotype). This report highlights the clinical features of SEIR and the role of insulin sensitizers like pioglitazone in the management of such patients.

**Conflict of interest:**None declared.

## INTRODUCTION

Syndromes of extreme insulin resistance (SEIR) represent an extreme end of a spectrum disorder of insulin resistance due a defect either in the insulin receptor or downstream in the insulin signalling pathway [Leprechaunism, Rabson-Mendenhall syndrome (RMS), Type-A syndrome, lipoatrophic diabetes], or auto antibodies to insulin receptor/insulin. Hyperinsulinemia and differential resistance to insulin action in different tissues and organs explain most of the manifestations of these disorders.

Leprechaunism is the most severe of these conditions leading to death usually within the first year of life. RMS is a less severe form which can be differentiated from leprechaunism by presence of teeth and nail abnormalities, coarse facial features, acanthosis, pineal hyperplasia and survival till adolescence (1). We present an adolescent with SEIR who had RMS phenotype with atrial septal defect (ASD). 

## CASE REPORT

**Figure 1:**
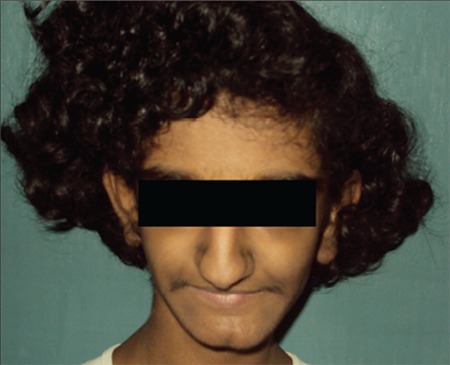
Facial profile of the patient showing pinched up nose, hollow cheeks, exuberant scalp hair, and increased terminal hair on face

**Figure 2:**
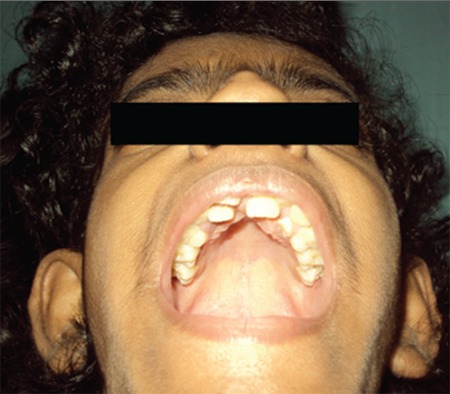
Upper jaw of the patient showing two rows of teeth

**Figure 3:**
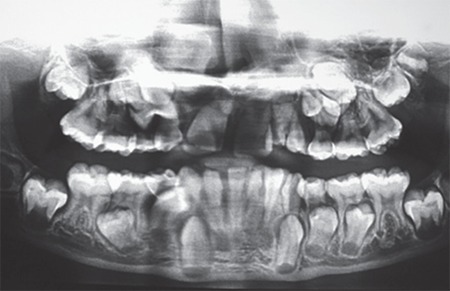
Orthopantogram showing two rows of teeth in upper and lower jaw

**Figure 4:**
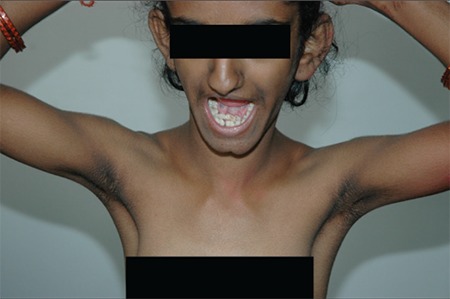
Profile of the patient showing two rows of teeth in the lower jaw, increased terminal hair on the face, along with acanthosis around the neck and axilla

A thirteen-year-old girl presented with poor height and weight gain, lack of development of secondary sexual features, primary amenorrhea, and skin hyperpigmentation. She was born to non-consanguineous parents at term with a birth weight of 1.5 kg and had delayed milestones, including delayed dentition. She had clitoromegaly since birth for which she underwent a reductive surgery at age 2 years. Examination revealed a bird-like facies, pinched up pointed nose, hollow cheeks (Figure 1), prominent mandible, abnormal dentition (Figures 2, 3), exuberant scalp and body hair, acanthosis over the nape of her neck, on the axilla, elbows and knuckles, a shrivelled skin mainly in her upper limbs and trunk (Figure 4). Her height was 130 cm (<3rd percentile; standard deviation score (SDS): -3.04), weight 20 kg (<3rd percentile; weight SDS: -2.32), and her body mass index was 11.83 kg/m2 (<3rd percentile). Mid-parental height was 147 cm (<3rd percentile; SDS: -2.28). Sexual maturity rating was prepubertal. External genital examination revealed an enlarged but mutilated clitoris (clitoral index: 60 mm2; normal <35 mm2).

**Figure 5:**
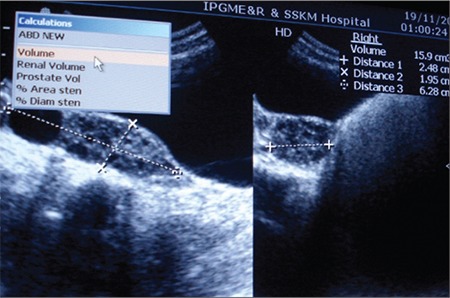
Ultrasonography of the abdomen showing bilateral polycystic ovaries with presence of a dominant cyst in the right ovary

**Figure 6:**
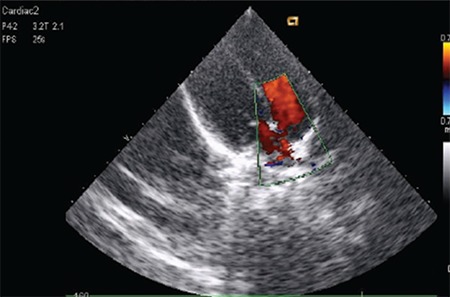
Echocardiography showing ostium secundum variant of atrial septal defect

**Figure 7:**
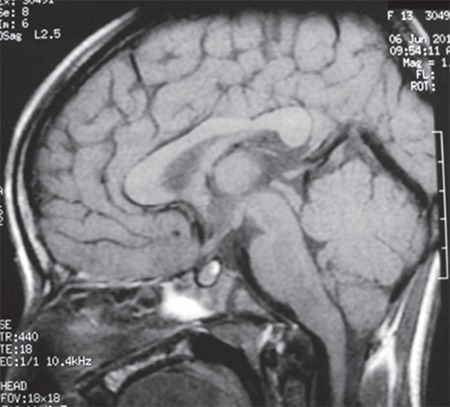
Magnetic resonance imaging of brain (T1W) showing normal pituitary and suprasellar region with normal pineal gland

Her karyotype was 46XX. She had fasting and post-glucose (following 35 g (1.75 g/kg) of glucose for a glucose tolerance test) hyperglycemia, hyperinsulinemia, and an increased C-peptide level (Table 1). Serum beta-hydroxybutyrate level was normal (Table 2) and urine ketones were negative. She had a raised serum testosterone level (0.067 nmol/L). Bone age was 13 years (Greulich-Pyle method). Abdominal ultrasonography showed normal kidneys and Mullerian structures, a liver with heterogeneous echotexture, and polycystic ovaries with echogenic stroma (Figure 5). Dual-energy X-ray absorptiometry (DEXA) showed reduced total body fat (11.3%; normal 15-25%). Echocardiography showed small ostium secundum ASD (Figure 6). Brain magnetic resonance imaging (MRI) showed a normal pineal gland (Figure 7).

Treatment was initiated with pioglitazone 15 mg/day, increased to 30mg/day after 6 weeks. Last evaluated after 1 year of initial diagnosis, her blood glucose is well controlled [fasting blood sugar glucose: 5.11 mmol/L; postprandial blood sugar glucose: 7.33 mmol/L; hemoglobin A1c (HbA1c): 0.069], she had no history of hypoglycaemia, her acanthosis had improved, and she had advanced into puberty (breast stage: B2, pubic hair stage: P3).

## DISCUSSION

Both alleles of insulin receptor are abnormal in RMS and these patients fail to respond to endogenous as well as exogenous insulin (2). The natural history of a patient of RMS is marked by fasting hypoglycaemia and postprandial hyperglycemia in the first year of life, followed by constant hyperglycemia (by 3-4 years age) and ketoacidosis usually by 6-7 years of age. Most patients die of severe intractable ketoacidosis by adolescence. It has been shown that there is a rapid fall in serum insulin levels in patients with RMS beyond 2 years of age, with maintained glucagon which may explain the ketosis. Infusion of very high doses of insulin (9.5 U/kg/hour) or high doses of insulin-like growth factor 1 (IGF-1) (1.6 mg/kg/day) have been shown to block ketone body production and control fasting hyperglycemia (2,3). High doses of metformin and glitazones (troglitazone, rosiglitazone) have been tried in patients with RMS with varied response (4). Recombinant methionyl human leptin (r-metHuLeptin) has been reported to be useful in controlling hyperglycemia, insulin levels, and HbA1c in patients with RMS and other lipodystrophy syndromes (4,5).

RMS was suspected in this patient because of the classical facial features, presence of exuberant scalp hair and hirsutism, typical dental abnormalities, growth retardation, acanthosis nigricans, lack of subcutaneous fat (lipoatrophy), hyperandrogenism, enlargement of external genitalia (clitoromegaly), extremely elevated levels of circulating insulin and also presence of other supportive biochemical parameters. Our patient was born small for gestational age, which is commonly seen in RMS. She had polycystic ovaries and fatty liver, and her lipid profile was typical of metabolic syndrome. Decreased total body fat on DEXA confirms the lipoatrophic phenotype. RMS is often associated with precocious puberty, in contrast to the findings of our patient. Presentation with delayed development and with mild hyperglycemia in our patient can be explained by the heterogeneity of mutations in the insulin receptor.This is the first report of occurrence of a congenital heart disease in a patient of SEIR with RMS phenotype. Heart diseases are uncommon in patients of lipoatrophic syndrome except for Berardinelli-Seip congenital lipodystrophy where hypertrophic cardiomyopathy is rarely reported in the third decade of life.

Counter-regulatory hormones have an important role in the pathogenesis of hyperglycemia in patients with RMS (6). Studies have shown normal to elevated growth hormone (GH) levels in patients with RMS (6,7) with transient improvement in metabolic parameters following hypophysectomy in a patient with RMS (8). Our patient had an elevated basal GH level but normal IGF-1 level, as also reported previously (9). However, recombinant GH and IGF-1 are not useful in increasing height in patients with RMS due to resistance to their action (4,9). Our patient has decreased whole body fat on DEXA, a finding which is expected in the broader group of lipoatrophic syndromes and also seen in RMS.

One of the limitations of this case report is the lack of study for mutation in the insulin receptor gene. Though uncommon, SEIR should always be considered in a child with suspicious phenotype, and the patient’s insulin resistance should be evaluated. Insulin sensitizers have an important role in the management of these patients, in contrast to insulin, which may be ineffective. To summarize, we presented an adolescent with SEIR, RMS phenotype, lipoatrophy, and ASD who responded to pioglitazone therapy. 

## Figures and Tables

**Table 1:**
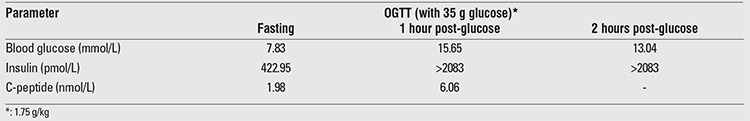
Changes in blood sugar, insulin, and C-peptide during the oral glucose tolerance test (OGTT)

**Table 2:**
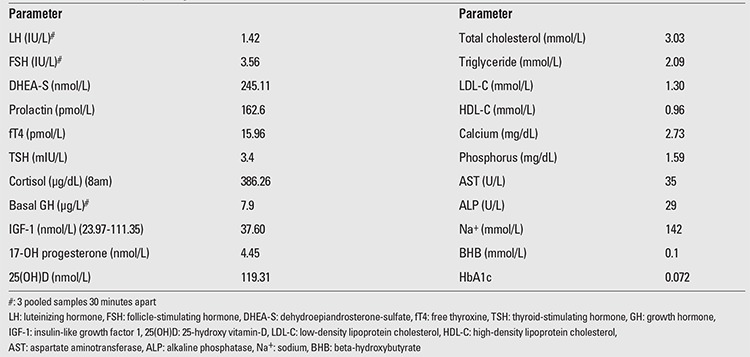
Baseline laboratory investigations
